# Nasal Morphology and Its Correlation to Craniofacial Morphology in Lateral Cephalometric Analysis

**DOI:** 10.3390/ijerph18063064

**Published:** 2021-03-16

**Authors:** Agnieszka Jankowska, Joanna Janiszewska-Olszowska, Katarzyna Grocholewicz

**Affiliations:** 1 Private Practice “Dental Clinic Jankowscy”, 68-200 Żary, Poland; agnieszkajankowska2301@gmail.com; 2 Department of Interdisciplinary Dentistry, Pomeranian Medical University in Szczecin, 70-111 Szczecin, Poland; katgro@pum.edu.pl

**Keywords:** nose, nose profile, cephalometry, orthodontics

## Abstract

Nose shape, size, and inclination influence facial appearance, but few studies concern the relationship between the nasal profile and craniofacial structures. The objective of this study was to analyze association of nasal cephalometric variables with skeletal structures, age, and sex. Cephalometric and nasal analysis was performed in 386 Polish orthodontic patients (aged 9–25 years). Student *t*-test and Mann–Whitney test were used to compare quantitative variables and Pearson’s or Spearman’s correlation coefficients—to find correlations. Soft tissue facial convexity angle correlates to Holdaway ratio, ANB (A-Nasion-B), and Wits appraisal. Nasal dorsum axis, nose length, nose depth (1) and nose depth (2), nose hump, lower dorsum convexity, and columella convexity increase with age. Nasal base angle, nasolabial angle, nasomental angle, soft tissue facial convexity and nasal bone angle decrease with age. Nasal base angle and nasomental angle are smaller in females. Thus, a relationship exists between nasal morphology and sagittal jaw configuration. Nasal parameters significantly change with age. Sexual dimorphism characterizes nasal bone angle and nasomental angle.

## 1. Introduction

The nose constitutes the most prominent part of the profile and occupies the most visible position in the face. It has a great influence on facial appearance and profile [1,2,3,4,5]. Thus, nasal balance and harmony are very important. Noses come in various sizes and shapes: upturned or straight, with or without a hump. Similarly, as a facial profile, the nasal dorsum can be classified as: straight, convex, or concave [6].

The nose, lips, and chin create facial harmony. The ideal nasal proportion requires a straight nasal dorsum with the dorsal cartilage and nasal tip cartilage above the nasal tip, forming the supratip break, and the alar rims 1–2 mm superior to the columella in the lateral view [2,4,5,7]. The main difference between an attractive and a below-average woman’s face is not in the proportion qualities of the nose, but in the relationship of the nasal and craniofacial measurements [5]. Moreover, the concept of an ideal nose is different among races, sexes, and ethnic groups [1,4]. Typical racial and ethnic differences in nasal morphology refer to the width, protrusion, and inclination of the longitudinal axis of the nostrils [5].

The nose shape in the cephalometric soft tissue profile is described by the nasolabial angle (NLA). By some authors, it is considered an excellent clinical and cephalometric parameter to reveal the anteroposterior position of the maxilla [1,8]. The NLA has two components: the inclination of the upper lip (lower nasolabial angle) and the upward nasal tip inclination (upper nasolabial angle) [1,9]. The inclination of the upper lip has a strong correlation with the amount of retraction of upper incisors, but nasal tip inclination has no correlation with incisor retraction [1,9,10]. Lo and Hunter [9] divided the NLA angle into its two contributing angles. (1) Nasal upward tip angle—the angle formed when the posterior columella point (PCm) (the most posterior point of the lower border of the nose) tangent is extended anteriorly to intersect with the Frankfurt horizontal plane/lower border of the nose to the Frankfurt horizontal plane; (2) the upper lip inclination—the angle formed by the PCm-Ls (labrale superius) line with the Frankfurt horizontal plane/inclination of the upper lip to the Frankfurt horizontal plane [1].

Previous studies evaluated the relationship between the nasal upward tip angle and vertical maxillary skeletal pattern. It was found that if an adult patient presents with an upturned nose, it might indicate that the maxillary plane is tipped anticlockwise [1]. Robinson et al. [6] analyzed lateral radiographs and proved that nasal shape followed the underlying skeletal pattern. Contrary results were obtained by Fitzgerald et al. [10], who found no correlation between soft tissue and skeletal measurements in the well-balanced profile.

Morphology of the nose shows a correlation with skeletal Classes. A pronounced elevation of the nasal dorsum and projection of the nasal bone is found in Class II subjects [6,11,12,13,14]. Class III subjects tend to have a concave dorsum and Class I subjects—a straight dorsum of the nose [12]. No relationship was found between the amount of nasal development and skeletal Class, the growth of the nose observed was relatively independent of the underlying skeletal hard tissue (skeletal Classes) [15].

Previous investigators stated that shape and size of the nose, and its inclination, have a significant influence on the orthodontic treatments plan. Excessive nasal growth in conjunction with extractions would probably have resulted in additional lip flattening and contributed directly to a poorly balanced profile [14]. Thus, orthodontic diagnosis and treatment planning should include nose evaluation and prediction of changes in facial aesthetics in a cumulative effect of the growth, development, and treatment [16].

Few studies have been found concerning the relationship between nasal profile and craniofacial structures. Thus, the purpose of this study was to analyze (1) correlations between nasal parameters and craniofacial skeletal structures; (2) correlations of nasal parameters with age (growth); and (3) sexual dimorphism of nasal parameters.

## 2. Materials and Methods

This study has been exempt from ethical approval, based on a written reply from the ethical commission. Sample size has been verified using an online power and sample size calculator (statisticalsolutions.net) assuming power of 0.8, level of significance at 0.05, and effect size equal to that calculated from a subsample of 100 patients.

After obtaining permission, 386 cephalograms of orthodontic patients aged 9–25 years were selected from the records of the Department of Radiology Pomeranian Medical University in Szczecin, based on the following criteria:

- Caucasian origin;
- Age 9–25 years;
- Good visibility of all cephalometric and nasal structures;
- Natural head position, the teeth occluded in the maximum intercuspation, relaxed lips;
- No craniofacial deformities;
- No fixed braces present at the time of the cephalogram.

**Figure 1:**
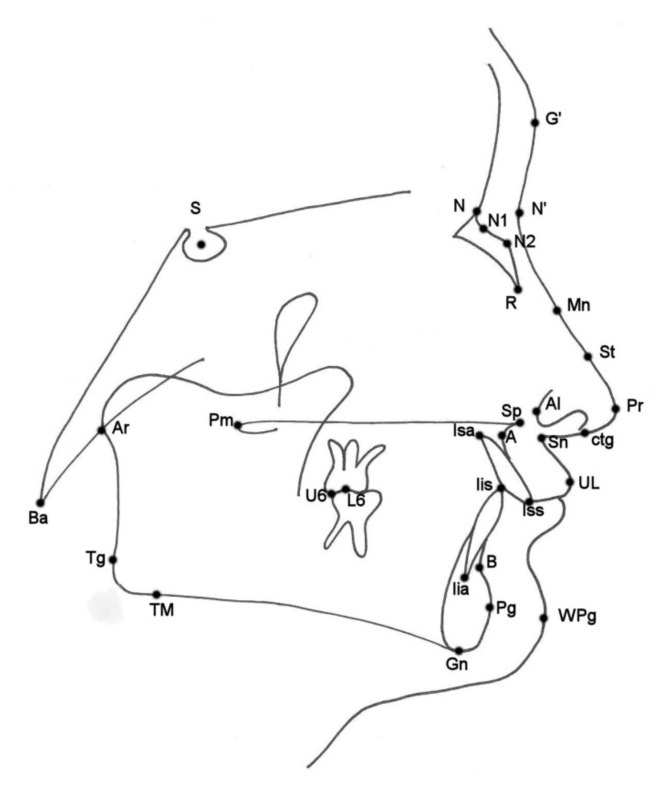
Nasal and cephalometric landmarks.

Cephalometric analysis, according to Segner and Hasund [17], was performed by the first author using a specialized computer software (Ortodoncja 8.0, Ortobajt, Wrocław, Poland). Analysis of nasal morphology was made according to Gulsen et al. [4] on acetate paper using a 0.5-mm pencil. The nasal and cephalometric landmarks used are presented in Figure 1 and described in Table 1. All cephalometric variables used are listed and described in Table 2.

Cephalometric and nasal analysis was repeated six months later, by the same investigator, in 100 randomly selected cephalograms. Repeatability of measurements was assessed using one-sided Wilcoxon test. The level of clinical significance has been set at 5 degrees for angular measurements and 2 mm for linear measurements.

Statistical analysis was performed using R software, version 4.0.3 [18]. Data distribution normality was assessed using Shapiro–Wilk test. The level of significance was established at *p* = 0.05. Comparisons of quantitative variables between the groups were made using Student *t*-test (for data of normal distribution) or Mann–Whitney test (otherwise). Correlations between quantitative variables were assessed using Pearson’s correlation coefficient (for data of normal distribution) or Spearman’s correlation coefficient (otherwise). The power of correlation was assessed according to the following schema [19]:

◦ |r| ≥ 0.9—very strong;
◦ 0.7 ≤ |r| < 0.9—strong;
◦ 0.5 ≤ |r| < 0.7—moderate;
◦ 0.3 ≤ |r| < 0.5—weak;
◦ |r| < 0.3—very weak.

## 3. Results

Sample size verification revealed that for the correlation coefficient between the SFC (Soft tissue facial convexity) angle and the H (Holdaway ratio) angle (0.763) a sample size of 11 subjects would be sufficient to show the significance, for the correlation coefficient between the NMA (nasomental angle) angle and the H angle (−0.517) the sample size yielded 37 and for the correlation coefficient between the NLA angle and the ANB (a point to B point angle) angle (0.247) 327 subjects would be sufficient to show the significance.

Wilcoxon one-sided test for repeated measurements revealed no differences between repeated measurements exceeding the level of clinical significance (5 degrees for angular measurements and 2 mm for linear measurements) for hump, NBA (nasal base angle), NMA, SFC. For N’–St (the axis of dorsum) and N’–Pr (nasal length) it was 1%, for nose depth (1) and NBoneA (nasal bone angle) it was 2%, for nose depth (2): 3%. For 1+:NA (°) (upper incisor angle) and 1−:NB (°) (Lower incisor angle) it was 4%, for 1+:NA (mm) and NBoneL (nasal bone length) it was 5%. For Pg:NB [mm] it was 6%. The highest percentage of discordant measurements (11%) was noted for the position of the lower incisors (1−:NB) (mm).

Characteristics of the study group, according to age, sex, and skeletal Class are presented in Table 3. Analysis of the data distribution normality are presented in Table 4. Cephalometric and nasal values of the study group are presented in Table 5.

**Figure 2:**
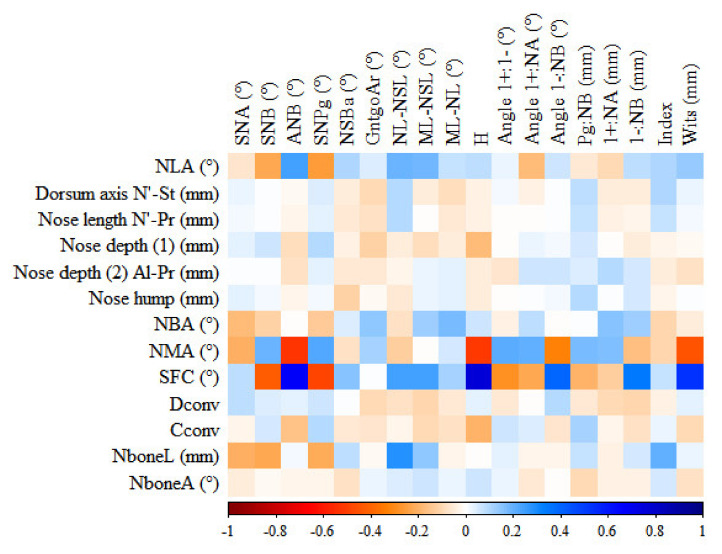
Correlation matrix for nasal and cephalometric variables.

The matrix of correlations between nasal and cephalometric variables is presented in Figure 2. The blue area shows strong positive correlation, the red area shows strong negative correlation. SFC angle shows the strongest correlations with Holdaway ratio (H), sagittal angle between maxilla and mandible (ANB), and Wits appraisal (Wits).

In the study group, a weak positive correlation with age was found for the following nasal parameters: dorsum axis, nose length, nose depth (1), nose depth (2), and a very weak positive for: nose hump (Hump), lower dorsum convexity (Dconv), columella convexity (Cconv). No correlation with age was stated for nasal bone length (NboneL). A weak negative correlation with age was found for NBA and a very weak negative correlation for: NLA, NMA, soft tissue facial convexity (SFC), and nasal bone angle (NboneA).

Significant differences were found between the sexes. Table 5 shows distribution of the study group according to nasal and cephalometric values referring to sex. Nasal base angle (NBA) and nasomental angle (NMA) were significantly smaller in females (*p* < 0.05). The average NLA is 113.32 ± 10.4 in females and 112.64 ± 13.34 in males, no significant differences were found between the sexes.

## 4. Discussion

Knowledge on correlations between the nasal parameters and the skeletal structures may help orthodontists and maxillofacial surgeons in diagnosing and treatment planning, for example the nose depth (1) and (2), nasal length, SFC, NMA, NBA, and hump determine the size and shape of the nose, whereas NLA angle is considered during the extraction treatment decision. The size of the patient’s nose is important for maxillofacial surgeons as they influence the occlusion as well as the profile of the patient’s face.

In the present study, the SFC angle showed a statistically significant positive correlation to H, ANB, and Wits, in accordance with the studies by Arshad et al. [2] as well as by Gulsen et al. [4], indicating a correlation to skeletal Classes. The SFC showed a weak negative correlation to SNB angle (Sella-Nasion to point B angle) (mandibular position), in agreement with the study by Gulsen et al. [4]. The very weak negative correlation between nasal bone length and SNA angle (maxillary position) confirms previous findings by Gulsen et al. [4] as well.

The negative correlation of the NMA angle to H, ANB, and Wits is in agreement with the results by Arshad et al. [2] as well as by Gulsen et al. [4]. The NMA angle is related to the skeletal Classes, to the upper and lower incisor inclination, to the maxillary and mandibular positions, as well as to maxillary inclination. Gulsen et al. [4] have found a significant correlation between the NMA angle and mandibular and maxillary position. In the study by Taha and Ahmed [20], the NMA angle was significantly higher in skeletal Class III compared to Class I and II.

The lack of statistically significant correlation between the Hump and skeletal Class is contrary to findings reported by Chaconas [13], who reported that Class II subjects proved to have a more pronounced nose hump, whereas Class I subjects tended to have a straighter nose [13].

The positive correlation between NLA angle and ANB angle (although very weak) is in accordance with the study by Gulsen et al. [4], but it is contrary to the results by Arshad et al. [2], as well as those by Taha and Ahmed [20]. A possible reason for this discordance may be the sizes of the study groups, e.g., insufficient number of subjects to determine statistical significance of a week correlation: Arshad et al. [2] (119 subjects), Taha and Ahmed [20] (90 subjects), Gulsen et al. [4] (262 subjects), present study (386 subjects). No significant differences concerning NLA angle were found between patients with skeletal Class I and other skeletal patterns (Class II/1, Class II/2, Class III) [21]. The very weak negative correlation between NLA and SNB (mandibular position) angles in the present study is consistent with the results by Gulsen et al. [4]. A positive correlation was found in the present investigation between NLA and mandibular inclination also increased, in accordance with the study by Gulsen et al. [4] on patients with a history of orthodontic treatment. However contrary to this are the findings by Nehra and Sharma [1], who reported no significant correlation. The reason could be that the study group was 190 Indian adults, who had undergone orthodontic treatment [1]. Burstone [8] reported that increased NLA indicated a maxillary retrusion and decreased—a maxillary protrusion. Contrary conclusions have been drawn by Gulsen et al. [4], who did not find any correlations between NLA and maxillary position, similarly as the present study.

Concerning nasal growth, the dorsum shape changes, especially during adolescence (between ages 10 and 14) and the hump of the nose appears during the adolescence period and is associated with positional changes of the nasal bone [11,22,23]. The nasal dorsum consists of upper and lower part. The angulation of the lower part of is closely associated with vertical growth changes of the tip of the nose [11].

Nasal development is almost completed in females by the age of 16 and in males by the age of 18 [1,3,12,22,23]. However, Meng et al. [3] stated that nasal growth in males continues above the age of 18. Nevertheless, most of the soft tissue development in women ceases at the age of 12 and in men—17 [15].

The nose is responsible for the total increase in soft tissue profile convexity with age [12,13]. The nose grows forward and downward and continues during maturation [2,3,12,13,22,23,24]. Compositely, this tendency increases nose prominence relatively to the facial profile [13,14,15,22,24,25,26]. However, female subjects have a smaller increase in nose depth than male subjects [3,12].

After the age of 14 years, the tip of the nose does not grow forward to the same extent as does the nasal bone and this results in nasal dorsum straightening or humping [22]. Subsequently nasal bone length is approximately 40–45 per cent of the total length of the nose [24]. In both sexes, the increase of growth in vertical dimension is much greater than the increase in anteroposterior dimension [24]. Interestingly, it was stated, that nasal growth occurred in all patients during orthodontic therapy, including those in whom skeletal growth had declined [14]. Nasal imbalance was intensified during orthodontic treatment [14].

During growth, soft tissue facial profile, excluding the nose, tends to remain relatively stable in its degree of convexity. However, when the nose is included in profile evaluation, the convexity of the soft tissue profile increases markedly with growth [12,15,23]. The present study is in agreement with these findings. Angular shapes and positional relationship of the nose, lips, and chin remain relatively constant throughout the developmental period for both sexes [15].

The increase of nose depth with age found is consistent with the study by Meng et al. [3], who proved that upper and lower nose height, as well as nose depth, showed most growth between 7 and 16 years, and females are characterized by a smaller increase in nose depth. Similar results were reported by Kumar et al. [27]. Moreover, nasal growth in men continued longer, after the age of 18 [3,13].

The increase of nasal dorsum with age is in agreement with the findings by Buschang et al. [11], who reported that nasal dorsum increases 10 degrees between 6 and 14 years, and changes are slightly greater during childhood. The increase in nasal length with age is consistent with the study by Chaconas [13] and Kumar et al. [27]. It was found that nasal length was correlated to other linear cephalometric measurements changing with age, for example mandibular length [4,13]. The length of the nose was negatively correlated to the angular measurement N’PrnP’ (nasomental angle) revealing that the length of the nose contributed to the convexity of the soft tissue profile [13].

In the present study, no statistically significant difference concerning NLA was found between white men and women, which is consistent with the papers by Fitzgerald et al. [10] and Hwang et al. [28]. Similar results were reported by Bagwan et al. [29], referring to Egyptian adults. However, contrary results were found by Magnani et al. [30] in young Brazilian black from 10 to 14 years, as well as by Taha and Ahmed [20] in Iraqi adults. Moreover, statistically significant sexual dimorphism of NMA angle is contrary to the studies by Taha and Ahmed [20], Hwang et al. [28], as well as by Lopatiene et al. [31], Aljabaa [32], and Kumar et al. [27]. The different findings may result from various sizes and age ranges of the groups analyzed. Taha and Ahmed [20] reported on 45 men and 45 women (age range: 18–25 years), Hwang et al. [28] reported on 15 men and 27 women (age range: 18–34 years), whereas Lopatiene et al. [31]—on 114 patients (age range: 14–16 years), Aljabaa [32]—on 32 females and 30 males (age range: 20–24 years), Kumar et al. [27] reported on 80 females and 80 males (age range: 8–16 years), while the present study reports on 229 females and 157 males (age range: 9–25 years).

The results of the present study referring to significant sex differences in nasal length, nose depth (1) and nose depth (2) and nasal hump are contrary to those reported by Gulsen et al. [4] on 262 Anatolian Turkish adults (age range: 18–30 years) as well as Arshad et al. [2] on 119 subjects of Pakistani origin (age range: 18–40 years). The present study is in accordance with the study by Aljabaa [32] on 62 Saudi subjects, (age range: 20–24 years), who found statistically significant sex differences in nasal length. The present study is contrary to this by Kumar et al. [27], who did not find significant differences in nose depth. Taha and Ahmed [20] observed no significant differences concerning nasal length and nose depth between the sexes. The significant difference for SFC angle between the sexes in the present study confirms the results by Gulsen et al. [4], however, is contrary to the study by Arshad et al. [2]. The different findings may result from age structure of the study groups or racial differences. The lack of significant sexual dimorphism referring to SFC angle is in accordance with the findings by Bagwan et al. [29].

Ethnic variability should always be taken under consideration. The NLA angle is smaller in Brazilian subjects of color than in white individuals [30]. It was also proved that this angle was significantly smaller in females [30]. However, black individuals showed similar angular measurements in both sexes [10].

NLA value in the literature has been presented in Table 6. All ethnic groups, presented in Table 6, had similar NLA angle in men and woman.

## 5. Conclusions

1. A relationship exists between nasal morphology and sagittal jaw configuration.
2. The following nasal parameters increase with age: dorsum axis, nose length, nose depth (1), nose depth (2), nasal hump, lower dorsum convexity, columella convexity. Parameters decreasing with age are: nasal bone angle, nasolabial angle, nasomental angle, soft tissue facial convexity, and nasal bone angle.
3. Sexual dimorphism has been found for nasal parameters: nasal bone angle and nasomental angle are significantly smaller in females.

## Figures and Tables

**Table 1:** Nasal and cephalometric landmarks.

Landmark		Definition
G’	Soft tissue glabella	Soft tissue point on the inferior part of the forehead between the eyebrows
N’	Soft tissue nasion	Soft tissue point—borderline between the forehead and the nose
Mn	Midnasale	Soft tissue point in the middle of the distance between the points N’ and Pr
St	Supratip	Soft tissue point halfway between the Midnasale point and the Pronasale points
Pr	Pronasale	Tip of the nose (soft tissue point)
Ctg	Columella	The most prominent point on the Sn-Pr curve, borderline between lower part of the nose contour and nasal tip
Sn	Subnasale	Soft tissue point between columella and upper lip.
UL	Upper Lip	The most protruding point of the upper lip
Al.	Alare	The most receding soft tissue point of the nasal alar curvature.
WPg	Pogonion	The most prominent point on the soft tissue chin
N	Nasion	The most receding point of the anterior surface of the frontonasal suture
N1	Nasion 1	The most receding point of the frontal curvature on the nasal bone
N2	Nasion 2	The most protruding point of the frontal curvature on the nasal bone
R	Rhinion	The most prominent and inferior point on the nasal bone
S	Sella	Centre the sella turcica
Sp	Spina nasalis anterior	The most protruding point of the nasal spine
A	Subspinale	The most posterior point on the anterior surface of the maxilla
Iss	Incision superius	Incisal edge of the most protruded upper central incisor
Isa	Incision superius apicalis	Apex of upper central incisor
B	Supramentale	The most posterior point on the anterior surface of the mandible
Iis	Incision infernus	Incisal edge of the most protruded lower central incisor
Iia	Incision infernus apicalis	Apex of lower central incisor
Pg	Pogonion	The most anterior point on the bony chin
Gn	Gnation	The most inferior bony point of the mandible
tgo	Tuberositas goniale	The point constructed of the intersection between the posterior and inferior edges of the mandible
Pm	Pterygomaxillary	The most posterior point of the nasal spine
Ar	Articulare	The point constructed of the intersection the lower contour of cranial base with the posterior contour of mandibular ramus
Ba	Basion	The most posterior and inferior point of the clivus
U6	Upper molar 6	Distal cusp of upper first molar
L6	Lower molar 6	Distal cusp of lower first molar

**Table 2:** Cephalometric and nasal variables.

	Abbreviation (unit)	Name	Definition	Interpretation
Nasal variables	N’–St (mm)	The axis of dorsum	Distance between the soft tissue nasion point and the supratip point	Length of the nasal dorsum
	N’–Pr (mm)	Nasal length	Distance between the N’ point and the Pr point	Total nasal length
	Nose depth (1) (mm)	Nose depth (1)	Perpendicular distance between Pr and the N’-Sn line	Sagittal position of the nose tip referring to the face
	Al–Pr (mm)	Nose depth (2)	Distance between points Al and Pr	Sagittal position of the nose tip referring to alar base
	Hump (mm)	Hump	Perpendicular distance between the axis of dorsum and its most prominent soft tissue point	Convexity of nasal dorsum
	NBA (°)	Nasal base angle	Angle between the G’-Sn line and the long axis of the nostril	Inclination of nasal base referring to the face
	NMA (°)	Nasomental angle	Angle between the axis of the dorsum and the Pr-WPg line	Relation between nasal dorsum inclination and chin position
	SFC (°)	Soft tissue facial convexity	Angle between the lines G’-Sn and Sn-WPg line	Profile convexity
	Dconv (mm)	Lower dorsum convexity	Perpendicular distance between the Mn-Pr line and its most prominent point	Convexity of the lower part of nasal dorsum
	Cconv (mm)	Columella convexity	Perpendicular distance between the Pr-Sn line and the most anterior point on the convexity of columella	Convexity of nasal base
	NboneL (mm)	Nasal bone length	The line constructed between the N point and the R point	Length of long axis of nasal bone
	NboneA (°)	Nasal bone angle	The posterior angle between the lines N1-N2 and N2-R	Curvature of the nasal bone
Cephalometric variables	SNA (°)	Sella-Nasion to point A angle	Angle between Sella-Nasion line and the Nasion-A line	Sagittal position of the maxillary alveolar process referring to anterior cranial fossa
	SNB (°)	Sella-Nasion to point B angle	Angle between lines Sella-Nasion and Nasion-B	Sagittal position the alveolar part of the mandible referring to anterior cranial fossa
	ANB (°)	A point to B point angle	Difference between SNA and SNB	Sagittal relation between maxilla and mandible
	SNPg (°)	Sella-Nasion to point Pg angle	Angle between the lines Sella-Nasion and Nasion-Pg	Sagittal position of the chin referring to anterior cranial fossa
	NSBa (°)	Cranial base angle	Angle between the lines Nasion-Sella and Sella- Basion	Inclination of clivus referring to anterior cranial fossa
	Gn–tgo–Ar (°)	Mandibular angle	Angle between the lines Gn-tgo and tgo-Ar	Angulation between mandibular corpus and ramus
	NL–NSL (°)	Maxillary base angle	The angle between the NL (maxillary base) line and Nasion-Sella line	Maxillary inclination to the anterior cranial fossa
	ML–NSL (°)	Mandibular base angle	Angle between the ML (mandibular base) line and Nasion-Sella line	Inclination of mandibular base to the anterior cranial fossa
	ML–NL (°)	Intermaxillary angle	Angle between the ML (mandibular) line and NL (maxillary) line	Inclination between the bases of maxilla and mandible
	NLA (°)	Nasolabial angle	Angle between the points ctg, Sn, UL	Relationship between the upper lip and columella
	H	Holdaway ratio	Angle between the lines UL-WPg and NB	Relationship between soft tissue profile and hard tissue profile
	1+1− (°)	Interincisal angle	Angle between the axes of upper and lower incisors	Inclination of upper and lower central incisors
	1+:NA (°)	Upper incisor angle	Angle between long axis of the most protruded upper central incisor and NA line	Upper central incisor inclination to maxillary base
	1−:NB (°)	Lower incisor angle	Angle between long axis of the most protruded lower central upper incisor and NB line	Lower central incisor inclination to mandibular base
	Pg:NB (mm)	Distance Pg-NB	Distance between Pg point and NB line	Sagittal position of the chin referring to alveolar part of the mandible
	1+NA (mm)	Upper incisor distance	Distance from upper incisor to NA line	Sagittal position of upper incisal edge to maxilla
	1−NB (mm)	Lower incisor distance	Distance from upper incisor to NB line	Sagittal position of lower incisal edge to mandible
	Index (%)	Lower face height index	Ratio between the middle and lower face heights	Lower face height in proportion to midface
	Wits (mm)	Wits appraisal	Distance between the perpendicular projections of points A and B on the occlusal plane	Sagittal relation between maxilla and mandible

**Table 3:** Characteristics of the study group (n = 386).

Feature		Values
Age	Mean ± SD	14.19 ± 3.58
	median	14
	quartiles	12–16
Sex	Females	229 (59.33%)
	Males	157 (40.67%)
Skeletal Class	I	173 (44.82%)
	II	138 (35.75%)
	III	75 (19.43%)

**Table 4:** Data normality (Shapiro–Wilk test, *p* < 0.05).

Variable	*p*-Value	Normality
Age	<0.001	No
SNA (°)	0.006	No
SNB (°)	0.001	No
ANB (°)	<0.001	No
SNPg (°)	0.001	No
NSBa (°)	0.516	Yes
GntgoAr (°)	0.332	Yes
NL–NSL (°)	<0.001	No
ML–NSL (°)	0.641	Yes
ML–NL (°)	0.132	Yes
H	0.803	Yes
Angle 1+:1− (°)	0.004	No
Angle 1+:NA (°)	0.211	Yes
Angle 1−:NB (°)	0.269	Yes
Pg:NB (mm)	<0.001	No
1+:NA (mm)	<0.001	No
1−:NB (mm)	<0.001	No
Index	0.972	Yes
Wits (mm)	<0.001	No
NLA (°)	<0.001	No
Dorsum axis N’–St (mm)	0.285	Yes
Nose length N’–Pr (mm)	0.47	Yes
Nose depth (1) (mm)	0.008	No
Nose depth (2) Al–Pr (mm)	0.001	No
Nose hump (mm)	<0.001	No
NBA (°)	0.475	Yes
NMA (°)	<0.001	No
SFC (°)	<0.001	No
Dconv	<0.001	No
Cconv	<0.001	No
NboneL (mm)	0.003	No
NboneA (°)	0.976	Yes

**Table 5:** Distribution of the study group according to nasal and cephalometric values.

Variable		Females	Males	Total	*p* ^1^
SNA (°)	mean ± SD	81.07 ± 3.76	80.13 ± 4.71	80.43 ± 4.68	0.022
	median	81.1	80.1	80.5	NP
	quartiles	78.9–83.6	77.2–82.9	78.0–83.1	
SNB (°)	mean ± SD	78.1 ± 3.88	78.04 ± 5.14	77.94 ± 4.71	0.572
	median	78.05	77.8	77.9	NP
	quartiles	75.38–80.53	74.7–81.1	74.9–80.6	
ANB (°)	mean ± SD	2.96 ± 3.08	2.09 ± 3.7	2.49 ± 3.5	0.031
	median	3	2.5	2.7	NP
	quartiles	1.1–5.2	−0.2–4.6	0.58–4.9	
SNPg (°)	mean ± SD	79.09 ± 4.06	79.07 ± 5.26	78.97 ± 4.82	0.473
	median	79.2	78.5	78.9	NP
	quartiles	76.4–81.6	75.5–82	75.9–81.7	
NSBa (°)	mean ± SD	129.81 ± 5.4	129.21 ± 5.49	129.5 ± 5.52	0.286
	median	129.5	129.4	129.4	P
	quartiles	126.1–133.4	125–133.1	125.6–133.3	
GntgoAr (°)	mean ± SD	125.92 ± 7.98	127.7 ± 7.73	126.77 ± 7.93	0.03
	median	125.2	127.9	126.20	P
	quartiles	120.5–131.6	122.4–132.8	121.2–132.3	
NL–NSL (°)	mean ± SD	7.8 ± 3.66	7.89 ± 3.88	7.86 ± 4.23	0.671
	median	7.7	7.9	7.8	NP.
	quartiles	5.6–10.2	5.7–10.3	5.6–10.3	
ML–NSL (°)	mean ± SD	32.27 ± 6.59	33.6 ± 6.85	32.9 ± 6.76	0.058
	median	32.3	34.3	32.9	P
	quartiles	28.2–36.9	28.6–38.4	28.3–37.5	
ML–NL (°)	mean ± SD	24.48 ± 6.37	25.97 ± 6.7	25.21 ± 6.66	0.069
	median	24.4	25.2	24.9	NP
	quartiles	20.3–28.6	21.5–30.3	21.1–29.5	
H	mean ± SD	11.35 ± 5.78	12.36 ± 5.74	11.55 ± 6.07	0.089
	median	11	12.9	11.7	P
	quartiles	7.3–14.7	8–16.6	7.3–15.8	
Angle 1+:1− (°)	mean ± SD	128.62 ± 11.74	128.1 ± 11.6	128.19 ± 13.85	0.545
	median	128.1	126.5	127.5	NP
	quartiles	121.2–135.9	120.6–134.5	120.9–135.9	
Angle 1+:NA (°)	mean ± SD	22.87 ± 8.31	24.57 ± 8.02	24.00 ± 10.5	0.047
	median	23.4	24.7	24.1	P
	quartiles	17.2–28.7	20.5–30	18.2–29.4	
Angle 1−:NB (°)	mean ± SD	25.6 ± 6.96	25.23 ± 7.21	25.35 ± 7.42	0.619
	median	25.7	25.9	25.7	P
	quartiles	20.9–30.52	21.5–30	20.8–30.3	
Pg:NB (mm)	mean ± SD	4.6 ± 5.95	6.58 ± 7.37	5.6 ± 6.76	0.028
	median	3.25	4.5	3.9	NP
	quartiles	1.08–7.7	1.3–11.9	1.1–9.7	
1+:NA (mm)	mean ± SD	11.6 ± 11.74	14.43±14.03	12.7 ± 12.8	0.031
	median	9.4	12.6	10.6	NP
	quartiles	3–18.7	4.7–22.2	3.4–19.5	
1−:NB (mm)	mean ± SD	12.83 ± 11.13	15.36 ± 12.11	13.8 ± 11.47	0.037
	median	10.15	14	11.55	NP
	quartiles	4.2–20.02	5–23.6	4.38–21.58	
Index	mean ± SD	79.69 ± 8.29	78.84 ± 8.62	78.52 ± 11.04	0.326
	median	79.5	79.1	79.1	P
	quartiles	74.4–85.6	72.7–84.6	73.5–84.8	
Wits (mm)	mean ± SD	0.03 ± 13.75	−3.97 ± 17.78	−1.28 ± 16.93	0.007
	median	0.8	−2.25	−0.3	NP
	quartiles	−4.67–5.62	−12.15–5.4	−8.7–5.8	
NLA (°)	mean ± SD	113.32 ± 10.4	112.64 ± 13.34	112.54 ± 12.46	0.589
	median	114.6	113.5	113.7	NP
	quartiles	106.1–120.3	105.8–121	105.7–120.5	
Dorsum axis N’–St (mm)	mean ± SD	44.01 ± 4.73	43.71 ± 5.71	43.81 ± 5.15	0.575
	median	44	44	44	P
	quartiles	41–47	40–47	40.5–47	
Nose length N’–Pr (mm)	mean ± SD	58.57 ± 6.15	58.4 ± 7.68	58.4 ± 6.82	0.811
	median	59	58.5	59	P
	quartiles	54.5–63	53–63	54–63	
Nose depth (1) (mm)	mean ± SD	23.8 ± 2.87	23.32 ± 3.16	23.6 ± 3.01	0.083
	median	24	23	24	NP
	quartiles	22–25.5	21–25.5	21.5 –25.5	
Nose depth (2) Al–Pr (mm)	mean ± SD	36.52 ± 3.53	37.12 ± 4.07	36.71 ± 3.79	0.136
	median	36	37	36	NP
	quartiles	34–39	34–40	34–39	
Nose hump (mm)	mean ± SD	−0.03 ± 1.42	0.17 ± 1.36	0.06 ± 1.39	0.118
	median	0	0	0	NP
	quartiles	−0.5–1	−0.5–1	−0.5–1	
NBA (°)	mean ± SD	96.5 ± 7.16	98.04 ± 7.71	97.07 ± 7.49	0.046
	median	96	97.5	97	P
	quartiles	91.5–101	93–103	92–102	
NMA (°)	mean ± SD	122.33 ± 6.08	124.46 ± 6.72	123.43 ± 6.55	0.018
	median	123	124	123.50	NP
	quartiles	118.5–126	120–128	120–127	
SFC (°)	mean ± SD	15.21 ± 7.26	14.79 ± 7.92	14.75 ± 7.90	0.579
	median	15	16	15	NP
	quartiles	11–20	11–20	11–20	
Dconv	mean ± SD	1.64 ± 0.97	1.54 ± 0.92	1.60 ± 0.95	0.435
	median	2	1.5	1.5	NP
	quartiles	1–2	1–2	1–2	
Cconv	mean ± SD	3.45 ± 1.15	3.67 ± 1.28	3.57 ± 1.26	0.098
	median	3	4	3.5	NP
	quartiles	3–4	3–4.5	3–4.5	
NboneL (mm)	mean ± SD	30.42 ± 4.51	30.84 ± 5.3	30.66 ± 5.02	0.465
	median	30	31	30.50	NP
	quartiles	27.5–33	28–34	28–33.5	
NboneA (°)	mean ± SD	148.02 ± 9.58	148.61 ± 10.21	148.25 ± 9.95	0.568
	median	147.5	149	148.50	P
	quartiles	142–154	141–156	142–155	

^1^ P = normality of distribution in both groups, *t*-test; NP = lack of normality in at least one group, Mann–Whitney test.

**Table 6:** NLA value in the literature.

Author, Year	Population	NLA Values		
		Men	Women	Total
Burstone [33], 1967	Caucasian adolescent	no data	no data	74 +/− 8.00
Fitzgerald et al. [10], 1992	white Americans	113.55 +/− 9.44	116.19	114.08 +/− 9.58
Arnett et al. [34], 1999	white Americans	106.40 +/− 7.70	103.50 +/− 6.80	no data
Fernandez-Riveiro et al. [35], 2003	Caucasians from Galicia, young adults	105 +/− 13.28	107 +/− 8.50	no data
Hwang et al. [28], 2002	European–American origin adults	112.05 +/− 9.86	109.71 +/− 7.60	no data
Hwang et al. [28], 2002	Korean origin adults	91.11 +/− 8.12	92.00 +/− 9.55	no data
Magnani et al. [30], 2004	Young Brazilian subjects of color	92.00 +/− 12.52	85.05 +/− 11.93	88.14 +/− 12.52
Scavone et al. [36], 2006	Japanese–Brazilian adults	108.40 +/− 10.76	110.10 +/− 8.97	no data
Nehra and Sharma [1], 2009	Indian adults	no data	no data	92.69 +/− 11.09
Dua et al. [37], 2010	Indian adults population	96.74 +/− 10.89	95.64 +/− 8.90	96.10 +/− 9.70
Anic-Milosevic et al. [38], 2011	Croatians	105.00 +/− 9.52	109.39 +/− 7.84	no data
Kandhasamy et al. [39], 2012	Komarapalayam adults population	116.51 +/− 8.01	115.701 +/− 4.00	116.10 +/− 10.00
Arshad et al. [2], 2013	Pakistani origin	100.55 +/− 14.52	98.87 +/− 15.76	no data
Paradowska-Stolarz and Kawala [40], 2015	white adult Poles—healthy patients	116.60 +/− 11.53	112.77 +/− 13.17	no data
Paradowska-Stolarz and Kawala [40], 2015	white adult Poles with any cleft (lip/ palate/both)	100.36 +/− 18.13	101.14 +/− 17.51	no data
Bagwan et al. [29], 2015	Egyptian adults	94.40 +/− 10.23	96.46 +/− 11.30	95.00 +/− 10.40
Aljabaa [32], 2019	Saudi adults	96.23 +/− 12.74	104.19 +/− 11.92	no data
Taha and Ahmed [20], 2020	Iraqi adults, skeletal Class I	97.93 +/− 9.75	101.73 +/− 12.15	no data
Taha and Ahmed [20], 2020	Iraqi adults, skeletal Class II	91.20 +/− 14.95	98.80 +/− 13.10	no data
Taha and Ahmed [20], 2020	Iraqi adults, skeletal Class III	95.33 +/− 10.75	101.93 +/− 9.81	no data
Perović et al. [21], 2020	Caucasian adults from Serbia, skeletal Class I	no data	no data	111.67 +/− 10.76
Present study	white adult Poles	112.64 +/− 13.34	113.32 +/− 10.40	112.54 +/− 12.46

## Data Availability

All data is a part of the manuscript.

## List of Abbreviations

NLA	nasolabial angle
PCm	posterior columella point—the most posterior point of the lower border of the nose at which it begins to turn inferiorly to merge with the philtrum of the upper lip (1).
Ls	labrale superius = UL—the upper lip point—the most anterior point on the upper lip (1).
NBA	nasal base angle
NMA	nasomental angle
Nose depth (1)	one of the two values of the nose depth
Nose depth (2)	(Al-Pr) one of the two values of the nose depth
Hump	nose hump
Dconv	lower dorsum convexity
Cconv	columella convexity
NboneL	nasal bone length
SFC	soft tissue facial convexity
NboneA	nasal bone angle
H	Holdaway ratio (H),
ANB	sagittal angle between maxilla and mandible
Wits	Wits appraisal
